# Estimating outcomes and cost effectiveness using a single-arm clinical trial: ofatumumab for double-refractory chronic lymphocytic leukemia

**DOI:** 10.1186/s12962-017-0071-x

**Published:** 2017-05-26

**Authors:** Anthony J. Hatswell, Gwilym J. Thompson, Penny A. Maroudas, Oleg Sofrygin, Thomas E. Delea

**Affiliations:** 1 University College London, London, UK; 2 GlaxoSmithKline UK, Stockley Park West, Uxbridge, UK; 3 Global Health Outcomes, GlaxoSmithKline, Uxbridge, UK; 4 Interdepartmental Group in Biostatistics, University of California, Berkeley, CA USA; 5 Policy Analysis Inc. (PAI), Brookline, MA USA; 6 BresMed, 84 Queen Street, Sheffield, S1 2DW UK

**Keywords:** Historical control, Cost-utility, Uncontrolled study

## Abstract

**Background:**

Ofatumumab (Arzerra^®^, Novartis) is a treatment for chronic lymphocytic leukemia refractory to fludarabine and alemtuzumab [double refractory (DR-CLL)]. Ofatumumab was licensed on the basis of an uncontrolled Phase II study, Hx-CD20-406, in which patients receiving ofatumumab survived for a median of 13.9 months. However, the lack of an internal control arm presents an obstacle for the estimation of comparative effectiveness.

**Methods:**

The objective of the study was to present a method to estimate the cost effectiveness of ofatumumab in the treatment of DR-CLL. As no suitable historical control was available for modelling, the outcomes from non-responders to ofatumumab were used to model the effect of best supportive care (BSC). This was done via a Cox regression to control for differences in baseline characteristics between groups. This analysis was included in a partitioned survival model built in Microsoft^®^ Excel with utilities and costs taken from published sources, with costs and quality-adjusted life years (QALYs) were discounted at a rate of 3.5% per annum.

**Results:**

Using the outcomes seen in non-responders, ofatumumab is expected to add approximately 0.62 life years (1.50 vs. 0.88). Using published utility values this translates to an additional 0.30 QALYs (0.77 vs. 0.47). At the list price, ofatumumab had a cost per QALY of £130,563, and a cost per life year of £63,542. The model was sensitive to changes in assumptions regarding overall survival estimates and utility values.

**Conclusions:**

This study demonstrates the potential of using data for non-responders to model outcomes for BSC in cost-effectiveness evaluations based on single-arm trials. Further research is needed on the estimation of comparative effectiveness using uncontrolled clinical studies.

## Background

Ofatumumab (Arzerra^®^, Novartis) is an anti-CD20 monoclonal antibody, which is under investigation in a range of diseases. In the first indication studied—chronic lymphocytic leukemia refractory to fludarabine and alemtuzumab [double refractory (DR-CLL)] in an early study 14 out of 33 (42%) patients had either a nodular partial response or a partial response [1].

Due to the lack of licensed (and established unlicensed) treatments in DR-CLL, the subsequent pivotal study, Hx-CD20-406, did not contain a control arm on the basis of clinical equipoise; as no other treatment had shown an effect in the condition, it was argued that it would have been unethical to deny patients the opportunity to experience results shown by ofatumumab in the previous study [2]. In Hx-CD20-406 ofatumumab was therefore administered to all patients with a dosing schedule determined by the dose finding program (a 300 mg loading dose, followed by up to 11, 2000 mg doses)—a study design that was discussed extensively by the FDA review [3]. As a result ofatumumab was administered to 59 patients with DR-CLL enrolled from 2006 to 2008, who at the time of the interim analysis showed median survival of 13.7 months (subsequently a further 36 patients were enrolled, with median overall survival increasing to 13.9 months). The evidence from this interim analysis of Hx-CD20-406 was deemed sufficient by regulators to grant ofatumumab marketing authorisation for CLL (subject to a follow-up study in this earlier line of disease), and in 2009 ofatumumab was granted a conditional licence for use by the European Medicines Agency, and accelerated approval by the US Food and Drug Administration [4, 5].

The lack of a control arm presents a challenge for researchers seeking to estimate the comparative effect of treatment compared to current practice in DR-CLL. Although an ongoing follow-up study of ofatumumab established the efficacy of CLL in untreated patients [6], no further studies were planned (or have been conducted) in DR-CLL. Where a relevant comparator is not included in the clinical study, indirect comparisons are recommended [7], but it is also possible to use an historical control [8]; often when using single-arm trials a group of untreated patients seen in a similar setting can be identified and comparisons made. An example of such an evaluation is seen in imatinib for the treatment of chronic myeloid leukemia, where the efficacy of interferon alpha was well characterised in published literature [8]. To account for differences between studies, different forms of adjustment can be applied which better match patient populations [9, 10].

In a systematic literature review for evidence in CLL, only one study was identified—Tam et al. [11] reported a case series of 58 patients with DR-CLL, treated with 19 different agents (including experimental agents, aggressive combination chemotherapy, and other high-cost therapies not routinely available in UK practice) at an international centre of excellence (the MD Anderson Center, TX, USA). As the treatments administered in this study were very different from the supportive care offered in the UK and the patients fitter (due to being able to tolerate the treatments received), this did not represent a suitable historical control for the patients in Hx-CD20-406. Equally as there are no randomized controlled trials, an indirect comparison was not possible.

Due to a lack of external data, we undertook a within-study comparison of all ofatumumab-treated patients and non-responders to ofatumumab, assuming the outcomes of ofatumumab non-responders were similar to outcomes seen with best supportive care (BSC). This approach assumed that non-responders received neither benefit or harm from treatment and therefore their path would be representative of that achieved by no treatment. A further assumption was that it was possible to control for differences in baseline prognostic factors between responders and non-responders using individual patient data such that the non-responders were better matched to the whole population. Here we describe the methods, results, and limitations of this approach, using the motivating example of ofatumumab for the treatment of DR-CLL.

## Methods

**Fig. 1:**
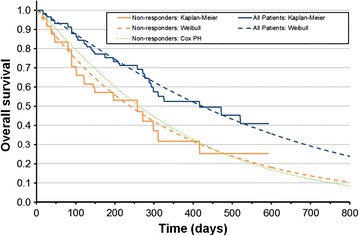
Overall survival for all patients vs. non-responders: Kaplan–Meier, parametric curve fits, and Cox proportional hazards model

CLL is a chronic disease and, in the absence of effective treatment, will continue to progress (CLL is not a condition where spontaneous remission is seen) [12, 13]. As no data was available on untreated patients, an assumption was made that BSC would therefore represent no response. To implement this assumption in modelling, outcomes for BSC were represented by those of the patients who did not show a response to ofatumumab in HX-CD20-406 (the primary outcome of the trial, defined according to the working group on CLL guidelines [14]). In the study, the median overall survival (OS) was 13.7 months, with the difference in OS between responders and non-responders is shown in Fig. 1; non-responders (n = 22) survived a median of 9.8 months, whilst the median OS of responders (n = 31) was not reached by the end of the 20-month study [15] based on landmark analysis at 12 weeks of patients who were able to be assessed. The comparison made in the model was therefore responders and non-responders to ofatumumab treatment, compared to non-responders (who were assumed to represent BSC). This is as response cannot be predicted a priori, so all patients must be treated to achieve the gains. We chose to use the interim data cut for the analyses presented here, as this represents the data that was available at the time decisions were made by both regulators and reimbursement agencies. This therefore gives more generalizable results, which also correspond with the published data [15].

To estimate progression free survival (PFS) and OS for ofatumumab, parametric survival functions were fitted to failure and progression time data from the all-patient group. PFS and OS in the BSC group were modelled by applying an estimated hazard ratio (HR) of survival between the non-responders and all-patient groups. The HRs were calculated based on a Cox proportional hazards model, in order to account for any important differences between baseline characteristics of the two groups i.e. factors that may predict response, leading to imbalances between the groups compared. In order to select the most appropriate parametric curve (from the lognormal, log-logistic, exponential, Weibull and Gompertz), the Akaike information criterion (AIC) and visual inspection were used. In all cases the Weibull curve provided the best fit by having the lowest AIC and providing a good visual fit to all parts of the Kaplan–Meier curve (i.e. not systematically under- or over-predicting for any section of the curve).

**Fig. 2:**
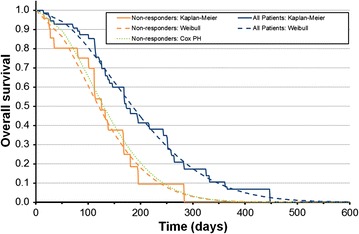
Progression free survival for all patients vs. non-responders: Kaplan–Meier, parametric curve fits, and Cox proportional hazards model

To model the non-responder group, the Cox regression included predictive covariates for sex, age, Rai stage, ECOG performance status, number of prior therapies, and years since diagnosis. This resulted in a change in the HR of 0.49, to an adjusted HR of 0.51 i.e. non-responders had had slightly less favorable prognostic characteristics, resulting in the adjustment. As an alternative to this approach a sensitivity analysis was also conducted using parametric curve fits to both arms (in place of the Cox regression which implicitly assumes proportional hazards). The resulting parametric curves and Cox regression results are shown in Figs. 1 and 2. All analyses were performed on the data available at the time (59 DR patients).

Ofatumumab was assumed to be administered in line with its licence, that is a 300 mg loading dose followed by up to 11 fortnightly doses of 2000 mg, administered until disease progression, death (if occurring prior to progression), or completion of the 24-week course. Patients who experienced disease progression were assumed to discontinue therapy. Whilst residing in a particular health state, patients were assigned the health state cost and the utility value linked to that health state. The effect of Grade 3 and 4 adverse events on costs was also included in the model, with the incidence taken from Hx-CD20-406 [15]. The effects of adverse events on utility were not included in the model.

**Fig. 3:**
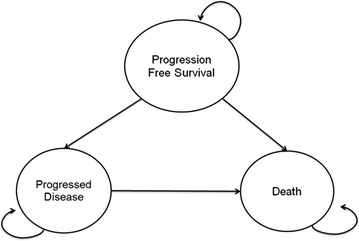
Economic model diagram

To extrapolate the results, a partitioned survival analysis model was constructed in Microsoft^®^ Excel 2003. The model had three health states: progression-free survival (PFS; the starting health state), progressed disease, and the absorbing state of death (Fig. 3). The proportion of patients in each health state over time is calculated using estimated survival distributions for PFS and overall survival (OS). The proportion in the progressed disease at any given time is calculated as the difference in OS and PFS at that time. This modeling approach has been used extensively in recent economic evaluations of novel cancer therapies. The model was then used to calculate the cost effectiveness of ofatumumab versus BSC, standard of care for patients with DR-CLL in the UK at the time of the evaluation. As previously discussed BSC was represented in the model by the non-responders to ofatumumab.

The analysis took the perspective of the UK National Health Service (NHS), with a base year for costs of 2010 [the year of the submission to the National Institute for Health and Care Excellence (NICE)]. The list price for ofatumumab was used in the analysis (£182 per 100 ml vial). Other costs were taken from NHS Reference Costs 2008/2009 and the Unit Costs of Health and Social Care [16, 17]. Utility data were taken from a time trade-off study of 60 members of the general public by Ferguson et al. [18] which used vignettes to derive preference weights for CLL health states—the only data available in this patient population. This study gave utility values of 0.650 [95% Confidence Interval (CI) 0.597–0.703] for PFS and 0.470 (95% CI 0.415–0.525) for post-progression survival. A 10-year time horizon was used, after which 97% of patients in the ofatumumab arm, and 100% of patients in the BSC arm were projected to be in the ‘dead’ health state. Both costs and benefits were discounted on a daily model cycle, at a 3.5% yearly discounting rate, in line with NICE (2008) and UK Treasury (2009) guidelines [19, 20]. Life years were not discounted. Table 1 shows a list of the key inputs to the model, along with the sources.

**Table 1:** Key costs and inputs to the economic model

Parameter	Value
Hazard ratio: overall survival, BSC vs. ofatumumab	1.8727
Hazard ratio: progression-free survival, BSC vs. ofatumumab	1.9608
Ofatumumab: cost of therapy initiation	£2047
Ofatumumab: cost per 100 mg vial	£182
Ofatumumab: concomitant medication cost	£104
Ofatumumab: cost per chemotherapy administration	£203
BSC: cost of therapy initiation	£1470
All therapies: cost per month of routine management	£158
Utility: progression-free survival	0.65
Utility: post-progression survival	0.47

*BSC* best supportive care

## Results

In the base case analysis (Table 2), ofatumumab was estimated to provide an additional 0.617 life years (1.494 vs. 0.877), and 0.301 additional quality-adjusted life years (QALYs) (0.770 vs. 0.469) compared to BSC. The estimated gain in OS consisted of additional 0.163 life years (2.0 months) in the PFS state, and an additional 0.453 life years (5.4 months) in the post-progression state. The results of the model closely matched those seen in Hx-CD20-406. Including extrapolation beyond the trial period, the model over predicted mean PFS (where data were mature) for non-responders by 5%, and under predicted PFS for all patients by 1%. Although the model fitted the OS data well over the observed period, due to the immaturity of the data and low patient numbers, no firm conclusions can be reached regarding the appropriateness of extrapolation.

**Table 2:** Disaggregated costs and outcomes of ofatumumab compared to best supportive care

	Best supportive care	Ofatumumab	Incremental
Costs			
Drug costs	£0	£35,081	£35,081
Administration costs	£0	£1834	£1834
Adverse event costs	£1665	£1339	−£326
Pre-progression healthcare costs	£2183	£3782	£1600
Post-progression healthcare costs	£908	£1906	£998
Total cost	£4756	£43,942	£39,186
Life years			
Progression-free life years	0.379	0.543	0.163
Post-progression life years	0.498	0.952	0.453
Total life years	0.877	1.494	0.617
Quality-adjusted life years (QALYs)			
Progression-free QALYs	0.244	0.348	0.104
Post-progression QALYs	0.225	0.421	0.196
Total QALYs	0.469	0.770	0.301
Cost-effectiveness ratios			
Cost per life year			£63,542
Cost per QALY			£130,563

*QALY* quality-adjusted life years

The incremental cost of £39,186 consisted mostly of drug cost (£35,081). Based on these results, the incremental cost-effectiveness ratio (ICER) was £130,563 per QALY, or £63,542 per life year gained. The difference between the cost per QALY and cost per life year can be attributed to the low utility experienced by patients, particularly in the post-progression health state (where utility was 0.470).

Results of sensitivity analyses (Table 3) show the model to be particularly sensitive to changes in the utility values used for health states—the wide confidence intervals from these estimates are due to the uncertainty in the original paper from which these were taken [18]. Using higher utility values decreased the ICER by £18,000, whilst lower utilities increased it by £82,000. The method of survival estimation for non-responders (to represent the BSC arm) was also important, with an increase in the HR of 20% for BSC reducing the ICER by £22,000, or including chromosomal deletions (clinically relevant predictors; however, with low numbers on each arm) in the Cox regression increasing the ICER by £15,000. When independent Weibull curve fits were used (instead of the Cox regression) the ICER increased by £13,000. However, it should be noted that the use of independent curve fits would not correct for any imbalance between groups at the baseline.

**Table 3:** Results of sensitivity analyses

Treatment	Total costs	Life years	QALYs	Incremental costs	Incremental life years	Incremental QALYs	Cost per life year gained	Cost per QALY gained
Base case results								
Best supportive care	£4756	0.877	0.469	–	–	–	–	–
Ofatumumab	£43,942	1.494	0.770	£39,186	0.617	0.301	£63,542	£130,563
Sensitivity analysis: utility values set to ‘following first-line treatment’ (progression-free survival 0.777 & post-progression 0.540)								
Best supportive care	£4756	0.877	0.551	–	–	–	–	–
Ofatumumab	£43,942	1.494	0.770	£39,186	0.617	0.219	£63,542	£112,067
Sensitivity analysis: utility values set to ‘following final treatment’ (progression-free survival 0.428 & post-progression 0.279)								
Best supportive care	£4756	0.877	0.294	–	–	–	–	–
Ofatumumab	£43,942	1.494	0.770	£39,186	0.617	0.476	£63,542	£211,918
Sensitivity analysis: independent curve fit used for control arm								
Best supportive care	£4884	0.952	0.497	–	–	–	–	–
Ofatumumab	£43,942	1.494	0.770	£39,058	0.542	0.273	£72,080	£143,402
Sensitivity analysis: alternative Cox regression including 17p and 11q chromosomal deletions								
Best supportive care	£4876	0.945	0.501	–	–	–	–	–
Ofatumumab	£43,942	1.494	0.770	£39,066	0.550	0.269	£71,076	£145,524
Sensitivity analysis: hazard rate on best supportive care increased by 20%								
Best supportive care	£4524	0.748	0.405	–	–	–	–	–
Ofatumumab	£43,942	1.494	0.770	£39,419	0.746	0.365	£52,837	£108,205
Sensitivity analysis: ofatumumab administration time doubled								
Best supportive care	£4756	0.945	0.405	–	–	–	–	–
Ofatumumab	£45,694	1.494	0.770	£40,938	0.617	0.301	£66,383	£136,399

*QALY* quality-adjusted life year, costs and QALYs discounted at 3.5%, *ICER* incremental cost effectiveness ratio

The only scenario the model was not sensitive to (showing only a 4% increase in the ICER) was a doubling of the cost of drug administration (both pharmacist preparation cost and chemotherapy administration cost). This was included in order to simulate any increased time that may be needed for the preparation of a new specialist product, which would not be in widespread use (in the NICE submission for ofatumumab, there were an estimated 13 patients per year in England and Wales) [21]. This increased the ICER by only £6000.

**Fig. 4:**
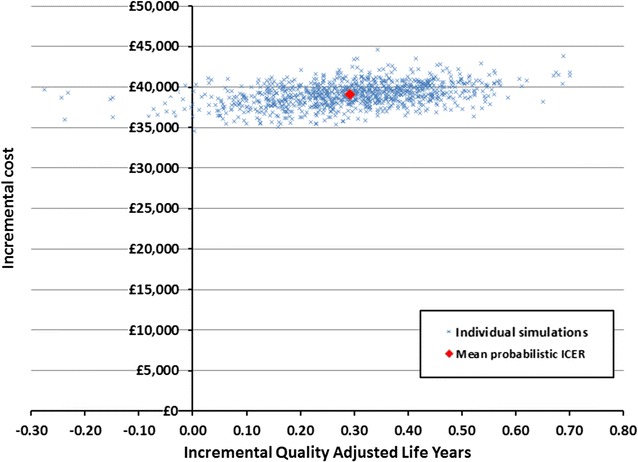
*Scatterplot* of probabilistic sensitivity analysis (1000 simulations)

A probabilistic sensitivity analysis (Fig. 4) of 1000 Monte-Carlo simulations demonstrated the majority of the uncertainty to be related to the magnitude of QALY gains, and not uncertainty in costs. This is due to the treatment course (the largest cost) being a fixed length and dosage; whilst there was substantial uncertainty in both the utility and clinical effectiveness estimates. In 2.5% of scenarios, ofatumumab was dominated by BSC (i.e. it provided worse outcomes and higher costs), which reflects the degree of uncertainty in the clinical evidence for ofatumumab.

## Discussion

The approach presented allows the estimation of cost effectiveness from a single-arm trial, where no historical control is available. However, there are a number of important limitations that cannot be addressed using the available data. The assumption implicit in the use of non-responders as a control arm was that the drug had no benefit or harm in these patients (which in this case is untestable due to the lack of control arm). If there was a disease-modifying benefit in non-responders (which did not reach the threshold for the definition of response), the effectiveness of ofatumumab versus BSC would be underestimated. The sensitivity analysis increasing the HR of BSC had a large impact on the ICER, showing the importance of the assumption that non-responders received no benefit: a 20% increase in the HR led to a 17% decrease in the ICER. It is therefore important to ensure the correct model is specified, and consider sensitivity analyses.

Conversely it also may be the case that the effectiveness of the treatment arm is overestimated when non-responders are used to estimate the outcomes for BSC. If responders had better prognostic indicators (either observed or unobserved), then it may be that patients who were responders would have performed better, regardless of treatment. Although the two groups were fairly well balanced in observable baseline characteristics, a lower proportion of responders exhibited deletion of chromosome 17p [15]. In a sensitivity analysis where the negative prognostic factors of chromosome 17p or 11q deletion were included in the Cox regression (used to estimate the HR for non-responders vs. responders) the ICER increased by 12%. However, the data used to build the Cox regression for this analysis were based on low patient numbers for chromosomal deletions (17p deletion n = 10 for responders and n = 7 for non-responders, 11q deletion n = 9 for responders and n = 15 for non-responders). This in turn highlights another limitation of the analysis; although attempts have been made to correct for any imbalances at baseline, responder status may have been linked to a characteristic not included in the Cox regression, or to an unobservable characteristic.

These two confounding factors (potential overestimation of the outcomes from BSC and overestimation of the treatment effect) may affect the analysis simultaneously, resulting in an overall bias of an unknown magnitude and unknown direction, which would affect not only time to event estimates, but also utility and resource use estimates. There is also a potential issue regarding artificially low estimates of standard errors; a subset of the all-patients group is used as a comparator, making the comparator group dependent on the treatment group, violating the assumptions needed for statistical inference of standard error estimation—further research on this topic would be required.

Results of the modelling in this case suggest that ofatumumab allows patients additional PFS and OS, based on the results of the Hx-CD20-406 trial and using non-responders to treatment as proxy for the outcomes of BSC. At typical UK willingness-to-pay thresholds, ofatumumab is not considered cost effective from the perspective of the NHS when analysed at list price [15]. Although, the manufacturer (at the time of the submission GlaxoSmithKline) proposed a patient access scheme in the submission to NICE, this was not evaluated in this paper as it was a confidential discount scheme.

The use of single-arm trial data also raises questions regarding the presentation of economic evaluations. In published appraisals, authors are encouraged to perform a sensitivity analysis, varying parameters within the 95% confidence intervals to generate a tornado diagram, and also to perform a probabilistic sensitivity analysis [22]. When using uncontrolled studies, performing only one-way sensitivity analyses and a probabilistic analysis does not truly characterise the uncertainty present in the clinical evidence. Although this issue is not unique to studies based on single-arm trials, the problem is more relevant as there is an underlying assumption that the clinical data used is suitable for comparison. In addition to analyses on the sensitivity of the model to parameter changes, scenario analyses are also needed where the fundamental assumptions underpinning the analysis are varied. In the ofatumumab model for example, it is not sufficient to test alternative curve fits to the control group, but the survival of the control group should also be explored (not seen as fixed data to which a variety of fits should be made).

Although there were a lack of options for the modelling of uncontrolled data, one option that it was not possible explore in this example (due to a lack of data availability) is to use data on patients who have failed on the previous line of treatment. In the case of ofatumumab, this would be patients who failed treatment in the alemtuzumab registration trial conducted by Fraser et al. [12]. These patients would have become double refractory on alemtuzumab failure, and thus been eligible for treatment with ofatumumab (had it been available). The treatment received would then have been ‘standard of care’ as ofatumumab was not available, effectively making this group an historical control. Although there are issues regarding data sharing between companies to allow such comparisons, a collaboration between Roche, GlaxoSmithKline, Sanofi and Boehringer Ingelheim and ViiV Healthcare has begun to provide researchers with access to data from completed clinical trials [23]. If this initiative is joined by other companies, researchers would be able to complete analyses that are not currently possible due to limitations in reporting in published papers—with patients being the ultimate beneficiaries.

In the process of modelling a single-arm trial, we recognise the limitations of previous approaches and also those of the approach we have used. Many questions remain regarding how best to perform cost-effectiveness analyses using single-arm trials; research is needed to show which methodological options are available, and suitable for use. The method proposed in this paper (of comparison to non-responders) can be tested in haematology clinical trials with control arms, and also simulation studies. Datasets where response has objective criteria (for example, blood counts) would allow a comparison between non-responders and placebo-treated patients. Further research on single-arm trials would also be valuable for other areas in which they are commonly used, such as orphan diseases. This is particularly important as even where published data can be used as an historical control, these can become invalidated by the introduction of new treatments. For example, the use of beta-interferons in multiple sclerosis, or targeted therapies in early-stage breast cancer have drastically changed the natural history of the disease and may render the use of previously published studies as historical controls invalid.

## Conclusions

In this paper, we describe an approach for presenting a cost-effectiveness comparison where comparative data are not available from either a control arm or an historical control. The method avoids the problem of naïve comparisons between single-arm trials with differences in settings and/or patient characteristics. While the proposed method has limitations (most notably the assumptions made regarding the disease course of non-responders, and independence of observations), it does allow the estimation of cost effectiveness in situations where otherwise it would be impossible. This, in turn, allows decision-making bodies (such as NICE) to use a best estimate in their deliberations, mindful of the uncertainty in analyses.

There is still a substantial need for research in economic analyses using outcomes from single-arm trials. Gaps include validation of the use of non-responders as a control arm in other datasets, and guidance on methods to estimate comparative efficacy using single-arm trials.
